# Myeloid sarcoma manifesting as generalized lymphadenopathy in a patient with myelofibrosis

**DOI:** 10.1002/ccr3.2473

**Published:** 2019-10-04

**Authors:** Christopher Trennepohl, Jonathan Sorah, Patrick Eulitt, Jonathan Galeotti, Joshua F. Zeidner, Nathan D. Montgomery, Catherine C. Coombs

**Affiliations:** ^1^ School of Medicine University of North Carolina Chapel Hill NC USA; ^2^ Department of Medicine University of North Carolina Chapel Hill NC USA; ^3^ Division of Hematology/Oncology Department of Medicine University of North Carolina Chapel Hill NC USA; ^4^ Department of Pathology University of North Carolina Chapel Hill NC USA

**Keywords:** hematology, myelofibrosis, myeloid sarcoma, oncology, pathology

## Abstract

Immunophenotyping is critical to the diagnosis of MS, as it can be difficult to differentiate from other diagnoses including lymphoma using conventional light microscopy.

## Graphical abstract

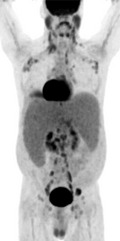

A 47‐year‐old male with no past history developed abdominal distension and fatigue, and was found to have anemia (hemoglobin 7.4 g/dL), thrombocytopenia (platelets 74 × 10^9^/L), and leukocytosis (WBC 33 × 10^9^/L) with 8% circulating blasts. Imaging showed marked hepatosplenomegaly and diffuse bulky lymphadenopathy (Figure 1).

**Figure 1:**
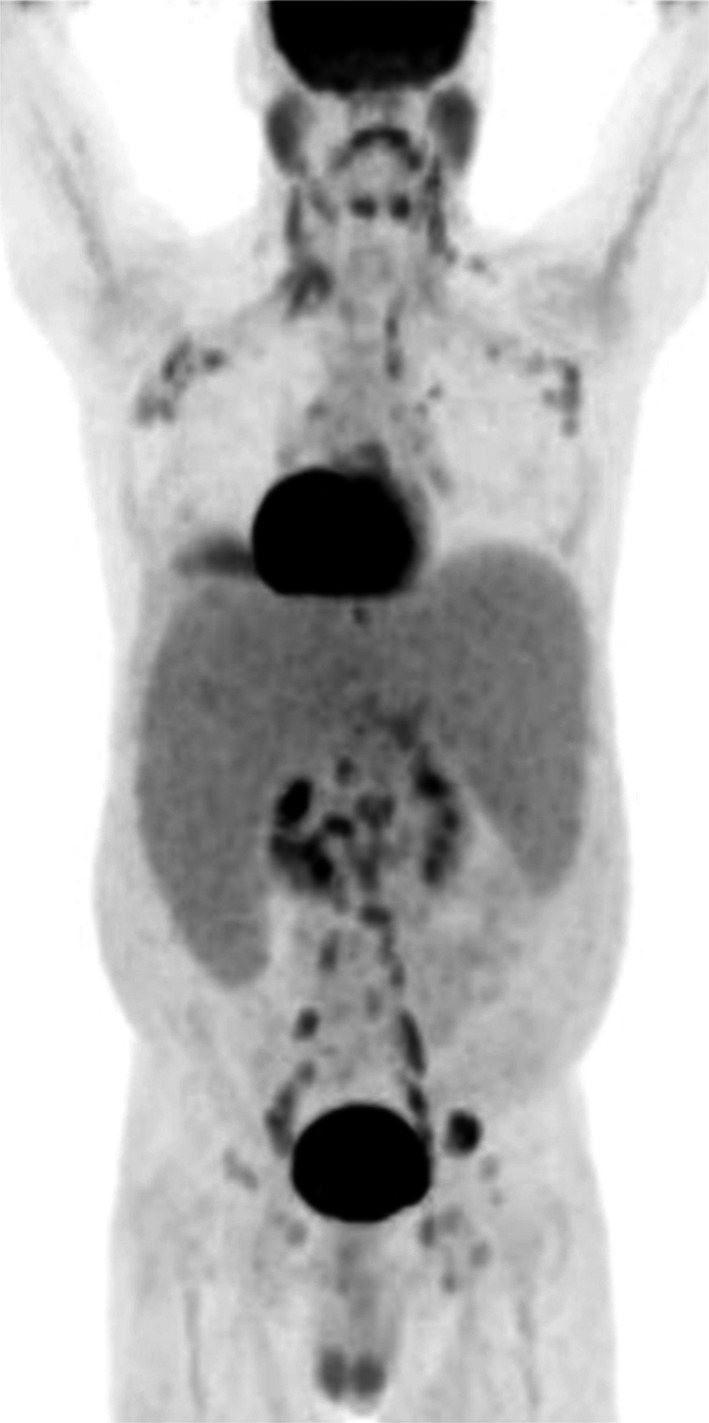
A PET/CT scan showing spleen length of 31 cm, liver length of 26 cm, and diffuse bulky lymphadenopathy

Bone marrow biopsy and clinical presentation were consistent with primary myelofibrosis (Figure 2). As lymphadenopathy is rare in PMF, he underwent axillary lymph node biopsy which demonstrated effacement by immature‐appearing cells (Figure 3). Flow cytometry showed an immature cell population (CD45 dim) that was negative for CD34, CD56, CD117, and MPO; strongly positive for CD4; and variably positive for CD10, CD33, HLA‐DR, CD68, CD123, and E‐cadherin, consistent with myeloid sarcoma (MS). The patient did not respond to induction chemotherapy with daunorubicin, cytarabine, and cladribine, but lymphadenopathy resolved after three cycles of decitabine and ruxolitinib. He progressed after six cycles, and was transitioned to azacytidine and venetoclax. He proceeded to allogeneic stem cell transplant, but died from veno‐occlusive disease on day +11.

**Figure 2:**
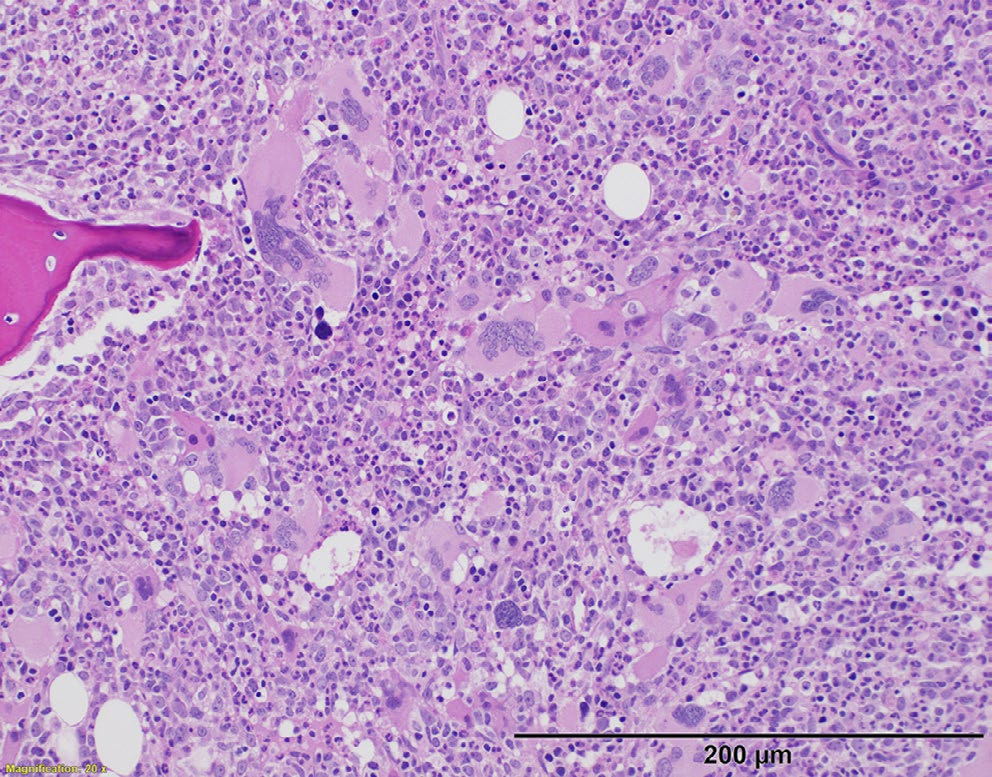
Bone marrow biopsy with hematoxylin & eosin staining, 10× objective magnification. Bone marrow is hypercellular (90%) with atypical megakaryocytes, fibrosis (MF‐2), and 1% blasts. Molecular testing demonstrated mutations in JAK2(V617F), NRAS(G12D), SETBP1(D868N and D874H), SRSF2(P95L), and TET2(Q916* and R1516*). Karyotype was normal on both bone marrow biopsy and lymph node biopsy

**Figure 3:**
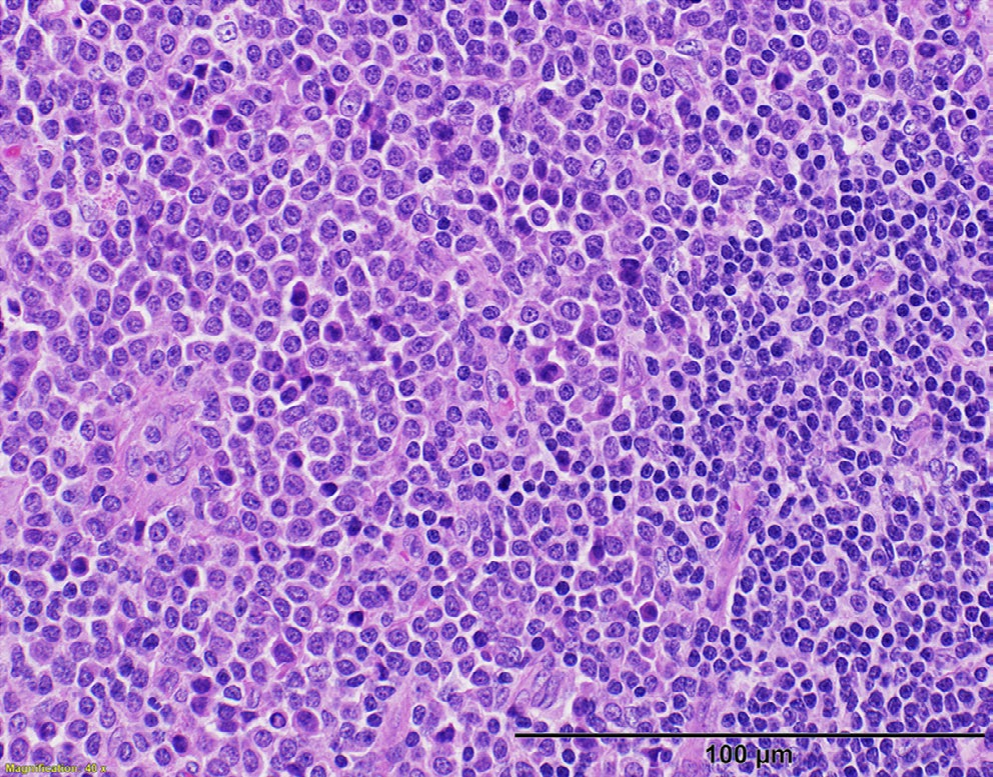
Lymph node biopsy with hematoxylin and eosin staining, 20× objective magnification. This shows an aggregate of small lymphocytes in the lower right representing a residual lymph node follicle; the remainder of the lymph node is effaced by immature cells. Karyotype was normal on both bone marrow biopsy and lymph node biopsy

While MS commonly affects lymph nodes,^1^ it rarely presents with diffuse involvement as in this case. Immunophenotyping is critical in diagnosing MS, as it can be difficult to differentiate from other diagnoses, including lymphoma, using light microscopy.^2^

## CONFLICT OF INTERESTS

CCC has received honoraria from Abbvie, H3 Biomedicine, and Pharmacyclics, has served as a consultant for Abbvie, has received institutional funding from Gilead, H3 Biomedicine, and Incyte, and has received travel funding from AROG and Incyte, JFZ has received honoraria from Celgene, Tolero, and Agios, has served as a consultant for Celgene and AsystBio Laboratories, and has received research funding from Merck, Takeda, and Tolero, and NDM received free sequencing from ArcherDx, and received prior research and travel funding from Ventana Biosystems, a subsidiary of Roche.

## AUTHOR CONTRIBUTIONS

CT: wrote the case report and cared for the patient. JS and PE: cared for the patient and contributed to writing. JG and NDM: reviewed the pathologic specimens and contributed to writing. JFZ: cared for the patient and contributed to revisions. CCC: cared for the patient and contributed to writing and revisions.
